# Reinforcement learning for mutation operator selection in automated program repair

**DOI:** 10.1007/s10515-025-00501-z

**Published:** 2025-03-15

**Authors:** Carol Hanna, Aymeric Blot, Justyna Petke

**Affiliations:** 1 University College London, London, England, UK; 2 Université de Rennes, Rennes, France

**Keywords:** Automated program repair, Machine learning, Mutation operators, Genetic improvement, Reinforcement learning

## Abstract

Automated program repair techniques aim to aid software developers with the challenging task of fixing bugs. In heuristic-based program repair, a search space of mutated program variants is explored to find potential patches for bugs. Most commonly, every selection of a mutation operator during search is performed uniformly at random, which can generate many buggy, even uncompilable programs. Our goal is to reduce the generation of variants that do not compile or break intended functionality which waste considerable resources. In this paper, we investigate the feasibility of a reinforcement learning-based approach for the selection of mutation operators in heuristic-based program repair. Our proposed approach is programming language, granularity-level, and search strategy agnostic and allows for easy augmentation into existing heuristic-based repair tools. We conducted an extensive empirical evaluation of four operator selection techniques, two reward types, two credit assignment strategies, two integration methods, and three sets of mutation operators using 30,080 independent repair attempts. We evaluated our approach on 353 real-world bugs from the Defects4J benchmark. The reinforcement learning-based mutation operator selection results in a higher number of test-passing variants, but does not exhibit a noticeable improvement in the number of bugs patched in comparison with the baseline, uniform random selection. While reinforcement learning has been previously shown to be successful in improving the search of evolutionary algorithms, often used in heuristic-based program repair, it has yet to demonstrate such improvements when applied to this area of research.

## Introduction

Fixing bugs remains a largely manual and tedious process that often requires more time than is available to software developers Weiß et al. (2007); Böhme et al. (2017). With the fast-evolving industry, this leads to the deployment of bug-prone products in an attempt to meet release deadlines Liu et al. (2021); Tassey (2002). Research in the area of automated program repair (APR) aims to address this issue by automating the process of finding suitable patches for bugs in software systems.

The classification of APR approaches differs in the literature. According to a recent survey Le Goues et al. (2019), the proposed approaches in the field of APR span across three main areas: constraint-based, learning-based, and heuristic-based techniques. Constraint-based approaches use the semantics of the buggy program to produce a constraint, then synthesise repairs that satisfy it Xuan et al. (2018). Learning-based end-to-end repair techniques predict patches for buggy programs by learning features of the faulty code sections as well as their correct (developer-written) fixes Chen et al. (2022). Among APR approaches, heuristic-based APR has seen the earliest and thus far most industrial uptake, including being first applied in the context of a management system for a medical application Haraldsson et al. (2017), or more recently being used for automated end-to-end repair at scale at Meta Marginean et al. (2019) or targeting frequently occurring bugs at Bloomberg Kirbas et al. (2020).

Heuristic-based APR uses search strategies, such as genetic programming Koza (1994) or local search Hoos and Stützle (2004), to navigate the space of software variants. These approaches require a test oracle for the buggy program to assess the correctness of the software variants. The oracle contains passing test cases and at least one failing test case that demonstrates the bug. As a first step, fault localisation techniques are employed to pinpoint the suspicious code sections. A program variant is created through the selection of a suspicious code location as well as a mutation operator to be applied at the location. The most common mutation operators are deletion, insertion, and replacement of fragments of code. A large population of candidate variants is produced through this process and validated against the test oracle to determine their fitness. The process is repeated until a suitable patch that passes all of the test cases is found.

In heuristic-based APR, the choice between the mutation operators in most approaches is random, causing the search to over-explore some operators that empirically lead to more failing software variants. As a result, more incorrect patches are produced and more resources are wasted which hinders the efficacy and efficiency of the technique. Smigielska et al. Smigielska et al. (2021) improved on this by implementing a uniform selection strategy where the probability for each operator to get selected is proportional to the size of its respective search space. Alternatively, Soto et al. Soto and Le Goues (2018) used developer bug fix history to determine a fairer distribution of operators.

A variety of mutation operators is necessary for a diversified search space. However, if the application of the mutation operators throughout the search process is not optimised, this can create many incompatible variants which will delay or even prevent finding a correct patch. While previous work Smigielska et al. (2021); Soto and Le Goues (2018) does address this concern, the probabilities for each operator remain predetermined and fixed throughout the search. Therefore, the online feedback of the search is not used to further tune the probabilities for the selection of each operator. Moreover, the optimal probabilities for the different operators are likely different for every software. We aim to tune the operators accordingly so that the search behaves at its best for the software that is currently being processed. Given that evolutionary computation is often used in APR as the search strategy, we drew inspiration from there. We found that augmenting a reinforcement learning agent to optimise the selection process of the mutation operators has been implemented for evolutionary algorithms Costa et al. (2008); Fialho et al. (2009, 2010); Thierens (2005); Murata and Ishibuchi (1996); Eiben et al. (2007); Pettinger (2002), differential evolution Sharma et al. (2019), and evolutionary programming Zhang and Lu (2008). To the best of our knowledge, this is yet to be applied for APR.

Our paper aims to tackle this gap in the literature by proposing a reinforcement learning based technique to inform the selection of mutation operators in heuristic-based APR to improve both the efficiency and efficacy of the process. We implemented two credit assignment techniques: average and exponential recency-weighted average credit assignment and four reinforcement learning-based mutation operator selection strategies: probability matching, adaptive pursuit, epsilon-greedy multi-armed bandit, and upper confidence bound. We experimented with two types of rewards: raw fitness values and fitness values relative to the parent. The activation point of the technique during the search process is also configured to best fit the context. We analysed the efficacy and efficiency of the different techniques to find the best strategy for guiding the search process in heuristic-based APR.

Our empirical results show that while the RL-guided mutation operator selection did generate more test-passing variants over the random one, it did not exhibit an improvement in the number of bugs patched. We hypothesize this could be due to the coarseness of the fitness function (Boolean value) which was not as effective in guiding the learning of the reinforcement agent. Although more fine-grained fitness functions have not shown positive results in this field Guizzo et al. (2021). Therefore, more work is needed to understand how to improve mutation operator selection during GI search.

To sum up, the contributions of our work are:

1. A novel reinforcement learning-based approach for mutation selection in heuristic-based program repair;
2. An extensible implementation of multiple selection techniques, credit assignment policies, reward calculations, and integration strategies in a state-of-the-art heuristic-based APR tool;
3. An evaluation on 353 bugs from a popular APR benchmark.

The rest of this paper is structured as follows: Section 2 presents the results of a literature review we conducted on the relevant topics, Sect. 3 presents background information, Sect. 4 presents our approach, Sect. 5 presents our research questions, Sect. 6 presents our methodology for answering the research questions, Sect.s 7 presents the results of the experiments, Sect. 8 presents a discussion of the observed results, Sect. 9 presents the related work we found through our literature review, Sect. 10 presents the threats to validity, and finally Sect. 11 presents the conclusions and future work.

## Literature review

To establish which mutation operator strategies would improve the efficiency and efficacy of search-based APR, we conducted a literature review. We focused on the two main areas that relate to this work: mutation operators and machine learning in the fields of automated program repair and genetic improvement.^1^

The literature review was conducted over four popular computer science search engines: IEEE Xplore, ACM Digital Library, ScienceDirect, and the DBLP Computer Science Bibiliography (details of the search are depicted in Table 1). All searches were conducted on 25/04/2024. The scope that we used for relevancy encapsulated all conference, workshop, and journal papers as well as PhD theses published by the search date from the fields of genetic improvement, evolutionary programming, and genetic algorithms.

**Table 1:** Literature review search results for relevant work on mutation operator selection

Keyword	‘mutation operators’		
Source	Filters	Papers	Relevant
		found	papers
IEEE Xplore	All Metadata	985	32
ACM Digital	Title OR	240	14
Library	Abstract		
ScienceDirect	Title,	898	12
	abstract or		
	author-		
	specified		
	keywords		
DBLP	Default	208	12
Total number of papers		2331	70
Distinct number of papers			65
Distinct number of papers on mutation selection			22
New Papers after snowballing round 1			14
New Papers after snowballing round 2			7
New Papers after snowballing round 3			3
Total number of distinct papers			46

**Keyword**	‘machine learning’ AND ‘program repair’		
Source	Filters	Papers	Relevant
		found	papers
IEEE Xplore	All Metadata	33	17
ACM Digital	Title OR	16	8
Library	Abstract		
ScienceDirect	Title,	3	1
	abstract or		
	author-		
	specified		
	keywords		
DBLP	Default	3	2
Total number of papers		55	27
Distinct number of papers			24
Distinct number of papers on mutation selection			20

The first keyword that we used was the exact phrase “mutation operators". After filtering for relevancy, we conducted a second filtering to find papers that involved mutation rate alteration. After we finished this primary search stage, we searched the bibliographies of the identified relevant papers (snowballing) and found 24 additional papers that met our paper selection criteria.

We then conducted a second search on the exact phrases “machine learning" and “program repair" using the same relevancy scope. To find the core papers here, we considered papers that use machine learning to improve the process of finding source-level repair candidates of existing software. The primary search for these keywords concluded with 20 papers. From these, the ones that apply machine learning based approaches to mutation selection are specifically for evolutionary computation.

Our literature review revealed there are four popular approaches for reinforcement based mutation selection, which we describe in the following section. None of these, however, have been applied to improve mutation selection strategies for improvement of existing software. We are thus the first to do so for the problem of automated program repair.

## Background

Next, we discuss the key ideas in heuristic-based automated program repair (APR) and reinforcement learning (RL) that are relevant as background for our proposed approach.

### Heuristic-based program repair

Heuristic-based program repair navigates the search space of software variants in order to find software that fixes a given bug Le Goues et al. (2019). The method is usually composed of three main stages. Given a buggy input program and its test oracle, the suspicious statements in the code are detected through the first fault localisation step. In the second stage, program variants are created by selecting mutation operators and applying them to the target locations to mutate them. Finally, the variants are all verified against the provided test oracle. A fitness function measures the viability of each variant based on the number of passing test cases and is used to guide the search strategy.

The two most commonly employed search strategies are local search Qi et al. (2014) and genetic programming Yuan and Banzhaf (2018). Local search is a heuristic approach to optimisation problems in which small and random local modifications are iteratively applied to find better solutions Hoos and Stützle (2004). As for genetic programming, a population of candidate programs is created through evolving the source code using steps inspired by biological evolution Koza (1994).

Our review of APR tools in the literature (Sect. 6.1) revealed that tools based on genetic programming are more common, thus we provide here more details on this search strategy. Traditionally, the genetic programming search strategy is compromised of 3 evolutionary steps: *selection*, *crossover*, and *mutation*. In the *selection* stage, the variants with the highest fitness in the population are selected. From there, the *crossover* transformation is applied to the population of variants in pairs. The result of crossing over two variants is a new offspring variant. In the *mutation* step, individual variants are mutated to create a second set of offspring variants.

Most commonly, a program statement is mutated through a random choice between one of the following three options: deleting the statement entirely, inserting another statement chosen from elsewhere in the code after the current statement, or replacing the current statement with a different one.

More recent heuristic-based APR research improve upon this by using developer behaviour information during the search Soto (2019), improving the fitness function Le (2016), addressing issues of overfitting Smith et al. (2015), and proposing different mutation operators Kim et al. (2013). However, the choice between the selection of the mutation options remains predominantly random with very few papers addressing improvements on this choice Smigielska et al. (2021); Soto and Le Goues (2018). We propose the augmentation of reinforcement learning strategies to optimise the selection of mutation operators during the search process.

### Reinforcement learning

In reinforcement learning (RL) Sutton and Barto (2018), sequential decision making is applied to problems where resources must be allocated between a finite number of conflicting choices. Given *N* different action choices where only partial historical information is known about each, the idea is to predict the best possible series of actions in order to maximise the cumulative outcome. Ideally, over time as more choices are made, the overall gain is optimised.

Balancing the exploration-exploitation tradeoff is at the core of RL techniques. *Exploitation* of actions that the algorithm has sufficient historical information about allows for a reliable estimation of the expected rewards. However, *exploration* is necessary in order to collect this information and optimise the selection of future actions. Choosing to exclusively exploit or explore actions will yield sub-optimal results.

RL-based approaches have been used to improve the mutation operator selection stage in evolutionary computation in the past Fialho et al. (2009); Maturana et al. (2009); Costa et al. (2008); Thierens (2005). In these works, the integration of such techniques is shown to outperform standard methods and optimise the selection of operators throughout the search. In this section, we detail the state-of-the-art RL algorithms that have been applied in this context as well as various variants for reward calculation and quality estimation from the literature.

#### Rewards

Multiple variations have been suggested for the calculation of the reward values that the actions receive in the RL algorithm. This can be the raw fitness itself, but usually it is the fitness improvement in reference to another individual. The reference individual can either be the offspring’s ancestor Fialho et al. (2009), the currently most fit individual in the population Davis (1989), or even the median fitness of the population Julstrum (1995). With this approach, an offspring that does not show an improvement is disregarded. In addition to just reflecting the fitness, the compass Maturana and Saubion (2008) technique also takes into account the population’s diversity. Beyond just considering quality (through fitness and execution time for example), they consider this additional dimension. Using these two different criteria of quality and diversity they are able to fine-tune the exploration-exploitation balance to allocate appropriate application rates to each operator.

#### Estimating action qualities

In this section, we discuss two techniques for estimating the qualities of actions Sutton and Barto (2018). In the first technique, the estimated quality for an action *A* at time step $t+1$ is the average of the rewards that the action has received until time step *t* (Equation 1). This approach is appropriate for stationary environments where the distribution of the rewards is not altered over time. Alternatively, exponential recency-weighted average accounts for dynamic environments where the reward probabilities are non-stationary. With this approach, the estimated quality for an action *A* at time step $t+1$ is calculated according to Eq. 2 such that $Q_A(0) = 1$ and $R_A(t)$ and $Q_A(t)$ are the reward and predicted quality for action *A* at time step *t* respectively. This equation introduces hyper-parameter $\alpha$, which controls the learning rate.

1 $$\begin{aligned} Q_A(t+1)&= \frac{\sum _{i=1}^{i=t} R_A(i)}{t} \end{aligned}$$

2 $$\begin{aligned} Q_A(t+1)&= Q_A(t) + \alpha [R_A(t) - Q_A(t)] \end{aligned}$$

#### Reinforcement learning algorithms

We present the state-of-the-art reinforcement learning techniques used for evolutionary strategies in the literature: probability matching, adaptive pursuit, and bandit-based algorithms.

*Probability matching (PM)* (sometimes also referred to as Thompson Sampling or Posterior Sampling) is a simple technique that is widely observed in biological processes. With this approach, the probability of taking an action mirrors the probability of getting rewarded for it. Specific implementation details differ in the literature; in this paper we follow Thierens et al. Thierens (2005) as their work applies to operator selection in genetic algorithms, often used in heuristic-based APR. To ensure that all actions continue to be explored throughout the search, they introduce a minimum probability value, $P_{\min }$. Given $P_{\min }$ and *N* actions, the probability for an action *A* at time *t* with the estimated quality $Q_A(t)$ gets calculated according to Eq. 3.

3 $$\begin{aligned} P_A(t) = P_{\min } + (1 - N * P_{\min }) \frac{Q_A(t)}{\sum _{i=1}^{i=N} Q_{\textrm{i}}(t)} \end{aligned}$$

*Adaptive pursuit* (AP) Thierens (2005) aims to solve the probability matching (PM) slow convergence issue by introducing a “winner takes all” strategy. As with PM, the actions are selected according to their probabilities. However, the probability of an action *A* at time step *t+1* is calculated according to Eq. 4. The action *M* is the action with highest quality value. This algorithm introduces a new hyper-parameter $\beta$ and a maximum probability value $P_{\max }$.

4 $$\begin{aligned} P_A(t+1) = {\left\{ \begin{array}{ll} P_A(t) + \beta [P_{\max } - P_A(t)] & \text {if A = M}\\ P_A(t) + \beta [P_{\min } - P_A(t)] & \text { otherwise} \end{array}\right. } \end{aligned}$$

*Bandit algorithms* are the state-of-the-art approach to mutation operator selection in genetic algorithms. In this paper, we experiment with two different variations: epsilon-greedy and upper confidence bound which have been shown successful in related work Fialho et al. (2009); Maturana et al. (2009); Costa et al. (2008).

The greedy bandit algorithm Sutton and Barto (2018) tends to favour exploitation of the best observed action thus far. Often as a result, the current best observed action keeps getting re-selected which prevents the algorithm from converging towards the best action. The epsilon-greedy Sutton and Barto (2018) improves on this by adding the option of exploration with a constant probability. With the epsilon-greedy algorithm, a random number between 0 and 1 is generated. If the generated number is less than $\varepsilon$, then an action is selected at random. Otherwise, the action with the highest quality (predicted reward) is greedily selected. Within bandit-based approaches the different action choices are referred to as *arms*.

The upper confidence bound (UCB) Auer et al. (2002) has been proven to optimise the cumulative gain and convergence rate. Given *N* actions, Equation 5 presents the UCB action selection at time *t* for action *A* where $Q_A(t)$ is the action’s estimated quality, *E* is a constant balancing exploration-exploitation tradeoff, and $n_{\textrm{A}}$ is the number of times that arm *A* has been played. The chosen arm at time *t* is the one that maximises the result of Formula 5. In the initialization phase, each arm is chosen once to collect initial awards. This ensures that $n_{\textrm{A}}$ is always larger than 0 and that the UCB algorithm formula can be applied.

5 $$\begin{aligned} Q_A(t) + E * \frac{\sqrt{\log \sum _{j=1}^{j=N} n _{\textrm{j}}}}{n_{\textrm{A}}} \end{aligned}$$

## Approach

In this work, we use reinforcement learning at the mutation operator selection stage of heuristic-based APR.

### RL-guided mutation operator selection

Our approach uses reinforcement learning to augment the mutation operator selection process in heuristic-based APR. Every mutation operator is associated with a score used to guide the overarching APR search process. This score is updated every time this operator is selected using the fitness of the associated variant, thus improving following selections.

We implement the four most commonly used strategies in heuristic numerical optimisation problems: selection based on probability matching, adaptive pursuit, epsilon-greedy bandits, and upper confidence bound bandits. We chose these operator selection strategies, in particular, as these are simple, yet shown successful in the context of genetic algorithms Thierens (2005); Costa et al. (2008) — a search strategy frequently used in heuristic-based APR. We are also the first to apply these in this context, thus it is yet unclear which ones, or variants thereof, would be successful. For each of the above techniques, we experiment with an operator’s reward as the raw fitness value or the fitness value relative to the parent and its credit being either based on exponential recency-weighted average or the average of the rewards (see Sects. 3.2.1 and 3.2.2). The variation in the credit assignment technique will inform whether the search process is stationary or dynamic.

This technique can be augmented into any heuristic-based APR tool that uses mutation operators. This is because we do not limit the number of actions (mutation operator options) to any specific value. We do, however, explore tuning the approach depending on the number of operators. We did this to investigate how to best apply the technique depending on the number of mutation operators the underlying tool implements.

### Credit assignment

When thinking about credit assignment in adaptive operator selection there are four main choices that must be made Fialho et al. (2009).

Firstly, **which type of reward will be used?** As this is the first paper to attempt reinforcement learning-based mutation operator selection in the context of automated program repair, we experimented with the two most simple approaches: the raw fitness value of the individual and the relative fitness of the individual in reference to its direct parent. For an action *A* at time step *t*, we calculate the relative fitness $R_A'(t)$ according to Eq. 6. This representation allows us to avoid negative numbers while still accounting for the exact level of improvement or deterioration in the offspring.

6 $$\begin{aligned} R_A'(t) = {\left\{ \begin{array}{ll} \frac{R_A(t)}{R_A(t-1)} & \text {if}\,R_A(t-1) \ne 0,\\ R_A(t) & \text { otherwise} \end{array}\right. } \end{aligned}$$

Secondly, **which operators to reward during the search?** Most commonly, only the operator that was used to create the offspring gets a reward. However, alternatives suggest rewarding the older ancestors of the offspring as well since they also had a part in its creation Fialho et al. (2009). Later work suggested that rewarding older individual’s operators in that way is less effective Barbosa and Medeiros (2000). Therefore, we only award the direct operator that was applied to create the offspring.

Thirdly, **how will the rewards accumulate throughout the search?** For each operator type, we can simply assign it its most recently received reward. We can also average all of the rewards that it has ever received. Since older rewards might be less relevant, Thierens et al. Thierens (2005) assign a window with a fixed size *N* where only the last N rewards contribute to the average. An improvement was made on this by Fialho et al. Fialho et al. (2009) to use an extreme-based credit assignment which is based on the assumption that infrequent large improvements in the fitness are more significant that frequent smaller ones Whitacre et al. (2006). We experiment with two simple strategies in this paper: using the average reward or the exponential recency-weighted average (see Sect. 3.2.2). Using these strategies, we are able to calculate the estimated quality of each mutation operator. Experimenting with both of these strategies will help us better understand how stationary/dynamic the search process is in APR.

Finally, **at which points in the search should the credit assignment occur?** An option would be to do so immediately after each operator is applied. This would mean that every time an operator is selected to create a variant, the variant’s fitness would be evaluated and used to update the credit of the operator. Another approach would be to do this in batches, i.e., after a constant or variable number of variants. We experiment with both options.

## Research questions

In heuristic-based APR the variety of mutation operators increases the chances of patching bugs Kim et al. (2013). However, this is at the cost of a much larger search space, and a slower search as a result. We explore whether an RL-based mutation operator selection solution can better guide the search and mitigate this slowdown.

**RQ1: Which credit assignment technique is suitable for mutation selection in heuristic-based APR?** The choice of RL credit assignment strategy depends on whether the expected reward values can change over the course of the search. We compare the performance of two main stationary and dynamic methods to determine the environment type in heuristic-based APR.

**RQ2: Which mutation operator selection strategy is best in heuristic-based APR?** RL has a long history of various selection strategies. We investigate the efficacy and efficiency of four different strategies that have been shown to be effective in similar contexts and compare with the standard operator selection technique in heuristic-based APR.

**RQ3: How does efficacy/efficiency of RL-based mutation selection in heuristic-based APR change with increase in the number of mutation operators?** The addition of more fine-grained mutation operators that target specific bugs could improve efficacy but impede efficiency of traditional heuristic-based APR by creating a larger search space. We investigate whether this issue can be fixed by our RL-based approach.

**RQ4: To what extent does RL-based mutation operator selection improve the bug fixing ability of heuristic-based APR?** Through online feedback of the search process, we hypothesise that the probability distribution of the mutation operators will be tuned to favour the more effective ones for the given buggy program, thus improving performance.

## Methodology

Next we describe our methodology for answering RQs. First, we conduct a pre-study, where we fine-tune learning rate $\alpha$ (see Equation 2) values for each of the four selection strategies. We use 10% of the dataset for these preliminary experiments. While the optimal dataset size for parameter tuning remains an open problem, running these experiments on the full dataset would be too costly and would risk overfitting to the benchmark.

To answer RQ1, we run each RL algorithm with both of the credit assignment techniques on a variety of real-world bugs from an APR benchmark. For each operator selection strategy, we analyse its efficiency and efficacy when combined with the various credit assignment techniques. We then compare each of the optimal combinations of operator selection and credit assignment identified from RQ1 with the baseline, i.e., without using RL. To answer RQ3, we explore the effect of changing the number of arms on the efficacy and efficiency rates of the proposed approach.

For the preliminary experiments and the first three research questions, we use the raw fitness value as the reward as it is the most simple strategy. We chose to assign credit to the operators at the end of each generation in batch at this stage to avoid too frequent updates that might overfit. Furthermore, most heuristic-based APR work relies on genetic programming which evolves populations of variants, already providing a natural division into batches.

For RQ4, we use the optimal settings of credit assignment and operator selection. In answering this question, we add experimentation for the reward types and integration strategy and compare our proposed approach with the baseline. We assess the quality of the patches by running the patched code on a second set of evaluation held-out test suites, which is a common strategy for patch evaluation in heuristic-based APR Motwani et al. (2022); Soto and Le Goues (2018); Soto (2019) (further details on patch correctness evaluation in Sect. 6.3).

We use the same measures for efficacy and efficiency to answer all of the research questions. Efficacy is measured by assessing the number of bugs for which a patch was generated, the frequency of such successful repair attempts for each bug, as well as patch quality, measured on held-out evaluation test suites. As for the efficiency measure we use the median and average numbers of individuals evaluated until a test-suite adequate patch is found.

### Tool

The field of APR has seen increasing growth in the last few years, with now tens of tools available^2^ to automate the task of bug fixing program-repair.org (2023). After a thorough review of the heuristic-based APR literature, we found that JaRFly Motwani et al. (2022) would be the best fit for our implementation. JaRFly is a novel open-source framework for search-based APR. It implements all three statement-level mutations that are used in GenProg Le Goues et al. (2012) and TrpAutoRepair Qi et al. (2013): append, delete, and replace. It also implements 18 PAR templates Kim et al. (2013) such as null checker and object initializer. JaRFly is well documented and very modular, making it an excellent candidate for extensibility.

When choosing a mutation operator to apply, JaRFly currently implements two options. The default is a uniform probability across all available mutations. They also allow for a probability distribution based on a probabilistic model. The model was created by mining open source repositories and analysing the frequency in which developers apply each of the available mutations Soto and Le Goues (2018). We chose to use the uniform distribution approach as the baseline because the results that were reported show that operator selection informed by the probabilistic model generated a smaller number of patches than the uniform distribution Soto (2019).

*JaRFly Modifications: * We implement a third reinforcement-based option in JaRFly that selects the mutation operator based on their current saved probabilities. These probabilities can be calculated according to any combination between the four operator selection strategies, the two credit assignment strategies, the two reward calculation techniques, and the two activation point variations. We implement each algorithm separately and depending on the mode with which the repair attempt was launched, the correct algorithm gets activated to set the probability values in the search process. We used the JaRFly fitness value as is in our implementation. The fitness value in JaRFly is calculated on the basis of the number of passing tests in the provided test suite.

Running the experiments revealed some bugs in the underlying code. We were able to locate five bugs that were causing uncaught exceptions in the execution of the code. These bugs are likely due to the changing version of external libraries that JaRFly depends on, e.g., in one of the cases this caused a change in the treatment of two-dimensional array identifiers. Patches for these bugs were added and can be found in our artefact. JaRFly does implement 18 PAR templates. PAR templates Kim et al. (2013) are fix patterns learned from human-written patches. Currently the most recent version has a bug that throws a NullPointerException within some scenarios of the application when 3 of the PAR templates generate variants, namely Parameter Replacer, Adder, and Remover. Therefore, we exclude these 3 PAR templates from all of our experiments. It is important to note that this exclusion was across all of the experiments that we ran and thus does not invalidate the results we obtained.

### Benchmark

We considered various benchmarks for evaluation through the overview that the program repair website program-repair.org (2023) provides. However, we chose Defects4J as it is currently the most comprehensive and popular dataset for evaluating Java APR tools Martinez and Monperrus (2016); Yuan and Banzhaf (2018) which allows us to be able to compare our results with the state-of-the-art. Additionally, Defects4J was used to evaluate the tool that we chose to implement our approach in, JaRFly Motwani et al. (2022), which allows for a direct comparison.

Our paper uses the most recent (2.0.0) version of Defects4J Defects4J (2023) for evaluation. Defects4J is a collection of real-world Java bugs from open-source repositories. Each defect in the dataset includes the defective version of code, its developer-fixed version, as well as test oracles. There are two types of tests that accompany every Defects4J bug: developer written tests as well as the infrastructure for generating automated tests using EvoSuite EvoSuite (2023) or Randoop Randoop (2023).

To fairly evaluate our approach, we used the exact Defects4J bugs that were used for the baseline JaRFly program. At the time that the JaRFly framework was evaluated, version 1.1.0 was used which consisted of 395 bugs. Given that the Mockito project was excluded in the JaRFly paper, we did not consider it. Finally, we omitted the four bugs that have since been deprecated. We evaluated our approach on the 353 remaining bugs. The JaRFly paper reports to be able to successfully repair 49 out of these bugs using the GenProg algorithm and an additional 15 active bugs using PAR templates. The breakdown of the bugs is presented in Table 2.

We chose not to extend our experiments to the entire 835 bugs of the latest Defects4J dataset, as it would have more than doubled the already very consequent computational budget involved. Indeed, with over 30,080 repair attempts and an average running time of about an hour per attempt (see Sect. 7) it amounts to about 3.5 years of continuous computation, or in our case multiple months of active cluster usage.

**Table 2:** Breakdown of the Defects4J bugs that were used in the JaRFly study Motwani et al. (2022) and patched using their tool

Project	Currently	Patched	Patched
	Active bugs	(GenProg)	(PAR)
JFreeChart	26	6	7
Closure compiler	131	15	20
Apache commons lang	64	9	12
Apache commons math	106	18	23
Joda-Time	26	1	2
Total	353	49	64

### Experimental set up

To answer our research questions, we ran the latest version of JaRFly. We used the default uniform selection strategy to get a baseline, before enabling the implemented RL-approaches.

*Search settings:* For each bug, 20 repair attempts were launched independently, to account for the heuristic nature of the underlying genetic algorithm. The search parameters were set to the same values as specified in the original paper of JaRFly Motwani et al. (2022). As such, each repair attempt was bound to 10 generations with a population size of 40. Since we were not able to run our experiments in the identical environment of JaRFly in terms of the hardware specifications, we eliminated the four-hour timeout per repair attempt and ran all experiments to completion of the 10 generations instead. JaRFly provides a replication package LASER-UMASS (2020) which includes the bugs that were successfully repaired as well as scripts for launching the tool to repair a specific bug. These scripts were used both for replicating their results and for conducting our experiments.

*Hyper-parameter settings*: The various reinforcement learning algorithms that we experiment with in this paper introduce hyper-parameters. Table 3 depicts the different parameters that are associated with the different algorithms (see Sect. 3.2 for more details on the parameters and formulas). The values of the parameters in the table are those used in the literature Thierens (2005); Fialho et al. (2009).

As for the learning rate $\alpha$ (from the exponential recency-weighted average formula — see Sect. 3.2.2), we conducted preliminary experiments to tune its value for each experiment type on a subset of bugs from the Defects4J dataset. The subset includes 5 diverse bugs which constitute just over 10% of the 49 bugs that the baseline in JaRFly successfully repaired. Each of these five bugs was the first in lexicographical order from those that the baseline in JaRFly correctly patched from each of the five projects (Table 2). The five bugs we used in the experiment were: Chart 1, Closure 102, Lang 10, Math 18, Time 19. For each of the four operator selection strategies, we conducted four experiments using four learning rate values for a total of 16 preliminary experiments. The value that we found in the literature for the learning rate in this context is 0.8 Thierens (2005). We experimented further with the learning rates 0.2, 0.4, and 0.6. Each repair attempt was repeated 20 times, to account for the heuristic nature of JaRFly’s genetic programming algorithm. Overall, in this preliminary study, we conducted 1600 repair runs.

We conduct statistical tests on the results of these preliminary experiments. Given that our data is not normally distributed (as per the Shapiro Test Razali and Wah (2011)), is continuous data, from independent samples, and compares over 3 groups, we utilized the Kruskal-Wallis Test McKight and Najab (2010).

**Table 3:** Hyper-parameter values for the PM, AP, epsilon-greedy, and UCB algorithms; *N*: no. of mutations

Name	Associated algorithms	Value
$P_{\min }$	PM, AP	$\frac{1}{2N}$
$P_{\max }$	PM, AP	$1 - (N - 1)*P_{\min }$
$\beta$	AP	0.8
$\varepsilon$	epsilon-greedy	0.2
E	UCB	10.0

**RQ1 and RQ2**: To answer the first 2 research questions, we activated our modified version of JaRFly with the 3 simple mutation operators from GenProg: insert, delete, replace (see Sect. 3.1) as these are the most commonly used and would provide a foundation to build on for evaluation. The evaluation for these 2 RQs was on the 49 bugs that the original JaRFly paper reports to repair using the GenProg algorithm excluding the 5 bugs that were used for the preliminary experiments for a total of 44 bugs. As explained in Sect. 6, at this stage we used the raw fitness value as the reward and assigned credits to the operators every generation.

**RQ3**: The experimentation for RQ3 was extended to include the 15 PAR templates that are implemented in JaRFly for a total of 18 mutation operators. Therefore, evaluation for RQ3 was conducted on all of the bugs that were repaired with either GenProg or PAR operators in JaRFly for a total of 64 bugs excluding the 5 bugs that were used for the preliminary experiments for a total of 59 bugs. Experimentation for RQ1-3 is limited to a small number of bugs to avoid over-fitting and find the best RL strategy for the ultimate task of repair which we investigate in RQ4.

**RQ4**: Given that we update weights after each generation, we note that it might not be enough time to evaluate all 18 mutation operators and learn optimal rewards. Moreover, several of the 18 operators naturally belong into groups, e.g., some manipulate parameter values, while others are concerned with bound checks. Therefore in RQ4, we activate the RL algorithm in the search using 3 different sets of arms for further evaluation:

1. 3 arms: 3 GenProg mutations
2. 18 arms: 3 GenProg mutations + 15 PAR templates
3. 7 arms: 3 GenProg mutations + 15 PAR templates aggregated into four groups.

The third set of arms groups together PAR templates that pertain to bugs within a similar category. We divide the PAR templates into four groups: functions and expressions (FunRep, ExpRep, ExpAdd, ExpRem), bound and null checks (NullCheck, RangeCheck, SizeCheck, LBoundSet, UBoundSet, OffByOne), casting and initialisation (CastCheck, ObjInit, CasterMut, CasteeMut), and multi-line edits (SeqExch). The mutations are chosen and applied as normal in the search algorithm. However, each operator’s credit gets assigned to its respective group. From there, an operator’s probability for selection is what was assigned to the arm of the group that the operator belongs to.

We experimented with two reward types: raw fitness and fitness relative to parent as well as two modes for assigning credit: credit assignment after every generation and after every mutation. We extend the evaluation to 353 bugs from the Defects4J benchmark.

We evaluate the correctness of the patches produced by our approach according to the same methodology used in JaRFly Motwani et al. (2022) for a fair comparison. The authors of JaRFly use two versions of the automated test generation tool EvoSuite EvoSuite (2023) to create held-out evaluation test suites. They created evaluation test suites for 71 defects from Defect4J that passed their criteria for statement coverage which are publicly available LASER-UMASS (2020). For each patch, if the defect had an evaluation test suite, we applied the patch to the defect and executed both sets of tests (v1.0.3 and v1.0.6) on the patched code. From there, we compared the quality scores of patches. The quality score for a patch is: $\frac{T_{\textrm{Pass}}}{T_{\textrm{Total}}}$ where ${\text {T}}_{\textrm{Pass}}$ is the number of tests that passed and ${\text {T}}_{\textrm{Total}}$ is the total number of test cases.

*Environment* Experiments were conducted on a cluster of 1028 compute nodes with varying specifications. RAM size ranged from 16GB to 375GB and local SSD disk space ranged from 80GB to 780GB. All CPUs were from the Intel Xeon processors range from a variety of generations with the number of cores ranging from 4 to 48. This did not affect the results as we ran all repair attempts to completion of the 10 generations.

## Results

Overall, a total of 30,080 independent repair attempts were conducted. Computation time for each repair attempt ranged, on average, from 45 minutes to 1.5 hours, up to 12 hours in longest runs. Overall, the experiments took multiple months of cluster usage equalling to 3.5 years of continuous computation.

**Table 4:** Preliminary experiment results: experiment type, learning rate ($\alpha$), percentage of repair attempts producing a test-suite adequate patch, number of bugs patched, average/median number of variants evaluated until first patch

Repair Type	$\alpha$	Success rate	Bugs patched (/5)	Avg. Variant	Med. Variant
Baseline	-	30%	4	177	171.5
PM	0.2	33%	3	154	131.0
	0.4	27%	3	141	80.0
	0.6	28%	3	178	93.0
	**0.8**	**34%**	**4**	**136**	**138.0**
UCB	0.2	36%	4	154	136.0
	0.4	34%	4	145	103.5
	0.6	36%	4	139	119.5
	**0.8**	**38%**	**4**	**155**	**117.0**
AP	**0.2**	**35%**	**4**	**127**	**79.0**
	0.4	33%	3	130	78.0
	0.6	28%	3	141	77.0
	0.8	31%	3	142	79.0
epsilon-greedy	0.2	24%	4	136	123.0
	**0.4**	**28%**	**4**	**133**	**97.5**
	0.6	30%	3	156	140.0
	0.8	18%	3	103	90.0

Highest/lowest values, results of our approach in comparison with baselines

### Preliminary experiments

**Fig. 1:**
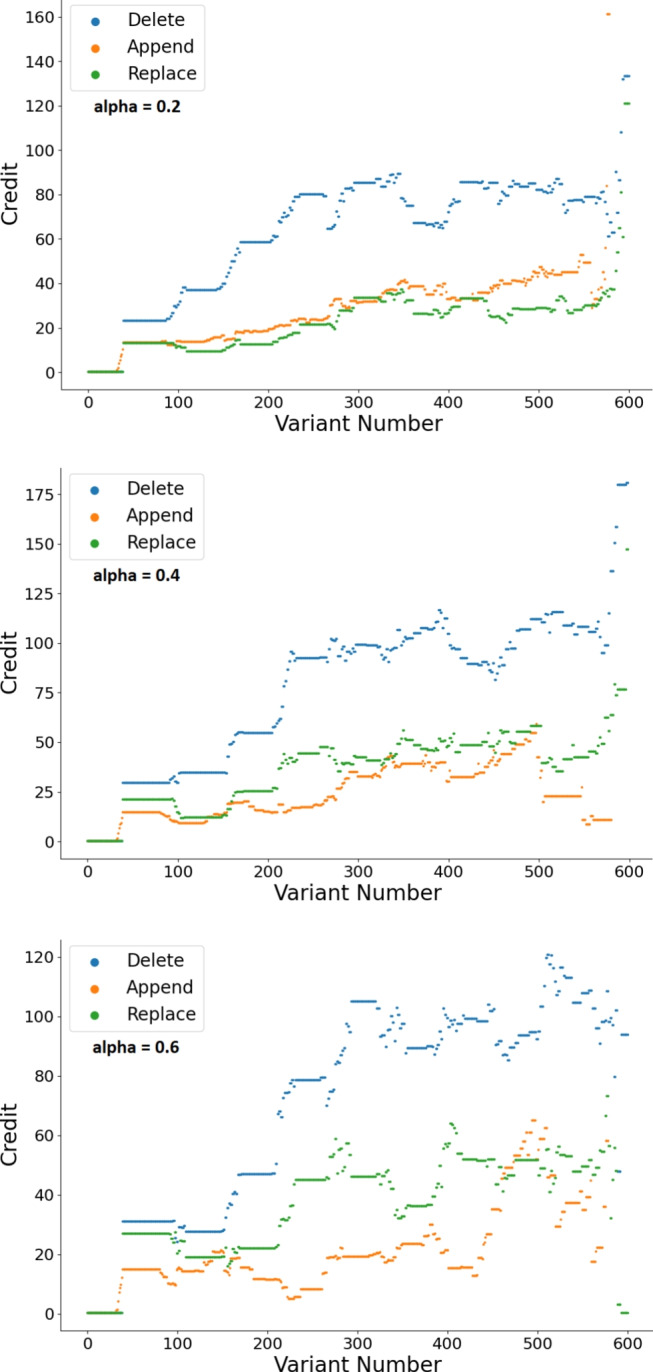
Mutation operators credit as a function of the variant number for the successful variant with the epsilon-greedy algorithm with varying learning rates (results are averaged over all runs). The credit values were sampled at the creation time of each variant (when a mutation is applied to a variant) before the selection stage, therefore the number of variants presented in the graph exceeds the population size. For the top suubfirgure, the trends present the results for the delete operator, append operator, and replace operator from top to bottom respectively. For the bottom two subfigures, the trends present the results for the delete operator, replace operator, and append operator from top to bottom respectively

The results of the parameter tuning preliminary experiments described in Sect. 6.3 are depicted in Table 4. We used these experiments to determine the optimal learning rate value for credit assignment using the exponential recency-weighted average for each operator selection strategy independently.

All 16 of the experiments that we set up at this stage had both higher successful repair attempts than the baseline and lower medians for the repair variant number. For each operator selection strategy, we chose the learning rate value that maximised the number of successful repair attempts. Given this criteria, for the probability matching and UCB algorithms we found that the optimal $\alpha$ value is 0.8. As for adaptive pursuit, it is 0.2 and for epsilon-greedy it is 0.4. These are the experimental set ups that we proceeded with.

We utilized the Kruskal-Wallis statistical test McKight and Najab (2010) on the success rates of the different settings to confirm our decision. When checking for a statistical difference between the 4 algorithms (PM, UCB, AP, Epsilon-Greedy), we found a p-value of 0.019 which confirms that using the different algorithms in this context results in statistically different success rates. However, when using the same test to check the statistical difference between the 4 learning rates (0.2, 0.4, 0.6, 0.8) on the same success rates, we found a *p* value of 0.874 which shows that there is not enough of a statistical difference between them.

As an example, Fig. 1 provides a visual representation of how the credits assigned to the different mutation operators changed throughout the search for the epsilon-greedy algorithm^3^. We can see that the learning rate 0.2 was too slow since the credits of the values stay somewhat stagnant, whereas with learning rate 0.6 it’s too noisy. This conclusion complements the results that we saw for the number of successful repair attempts for this experiment, in which learning rate 0.4 was optimal.

**Table 5:** RQ1 & RQ2. Repair attempts with average and exponential recency-weighted average credit assignment

Repair type	$\alpha$	Success rate	Bugs patched (/44)	Avg. Variant	Med. Variant
*Average credit assignment*					
**PM**	–	43.1%	38	93	62
**UCB**	–	47.0%	36	120	84
AP	–	44.8%	35	89	49
**epsilon-greedy**	–	48.3%	39	107	101
*Exponential recency-weighted average credit assignment*					
PM	0.8	43.3%	35	102	68
UCB	0.8	43.1%	35	115	73
**AP**	0.2	44.0%	37	95	63
epsilon-greedy	0.4	45.2%	40	107	70

Highest/lowest values, results of our approach in comparison with baselines

### RQ1: Best credit assignment technique

The results of the experiments with average and exponential recency-weighted average credit assignment are presented in Table 5. Interestingly, we can see that in all of the experiments the success rate of the repair attempts is higher with the average credit assignment than with the exponential recency-weighted average credit assignment. Moreover, we can see that for PM and UCB, the number of unique bugs that were fixed is higher as well. Therefore, average credit assignment is best suited for PM, UCB, and the epsilon-greedy algorithms whereas for AP, the exponential recency-weighted average is better.

We investigated the discrepancy for the unique number of bugs patched in the adaptive pursuit results and found that there were 3 bugs that were successfully patched in combination with the exponential recency-weighted average credit assignment that were not patched in combination with the average credit assignment. However, for these bugs, only 1 or 2 repair attempts successfully generated patches. i.e., their patches are harder to find in the search space. AP with the average credit assignment might have failed to produce these patches due to limiting the number of repair attempts to 20 in our experiments. We can thus conclude that the search process in heuristic-based APR is stationary.

**RQ1**: Average credit assignment is best suited for heuristic-based APR. Thus, the search process in heuristic-based APR is stationary.

**Fig. 2:**
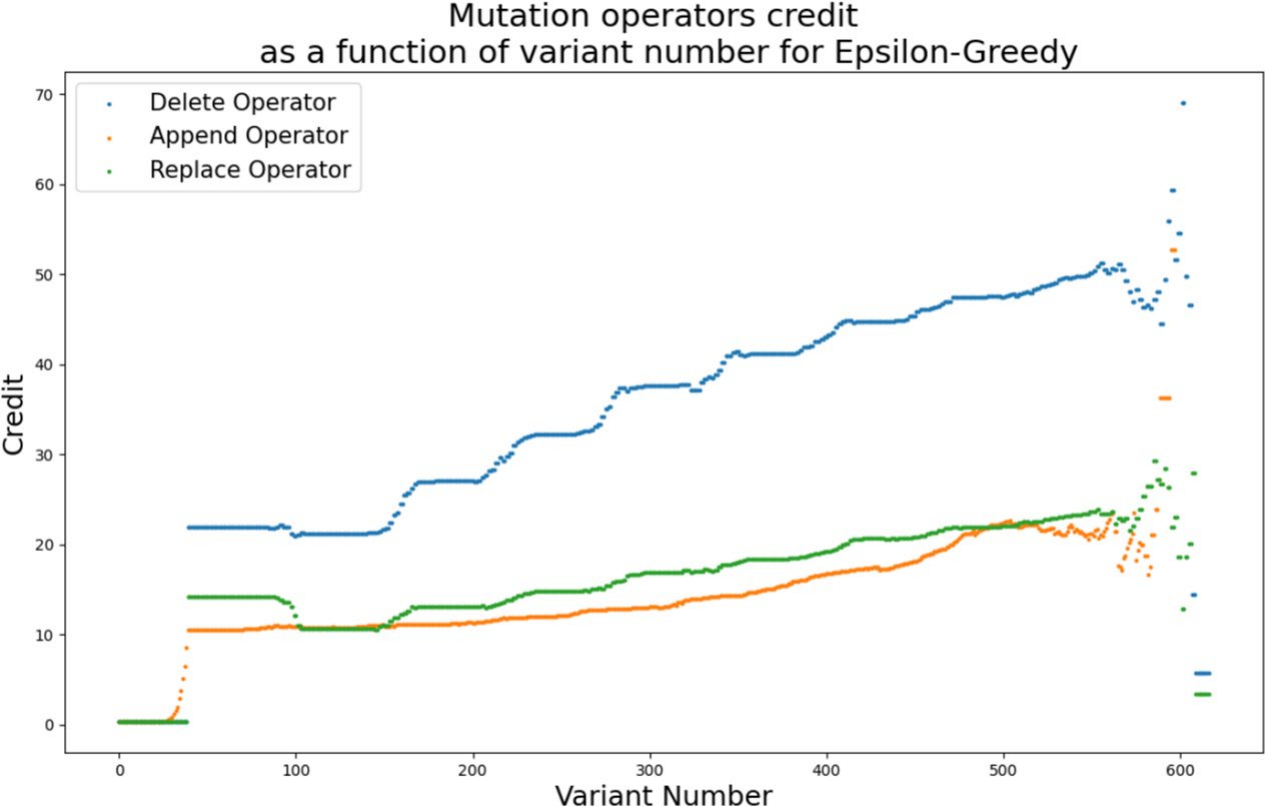
Trend of the credit assigned to mutation operators as the variant number increases for average credit assignment and epsilon-greedy operator selection. The trends present the results for the delete operator, replace operator, and append operator from top to bottom respectively

### RQ2: best operator selection technique

To fairly compare with the baseline in answering RQ2, we tried to replicate the baseline results from the JaRFly paper by running the 49 bugs (Sect. 6.2) that the authors report to generate patches for using the GenProg setting excluding the 5 bugs used in the preliminary experiments for a total of 44 bugs. Our results are presented in Table 6. When attempting to replicate the results, we were only able to generate a patch for 38 out of the 44 bugs. Since we used the exact search parameter settings and seeds that were used in JaRFly, we think that the discrepancy is likely due to the removal of the 3 PAR templates (as explained in Sect. 6.1) as well as mismatched versions (see details in Sect. 10), and heuristic nature of the search. The latest versions of JaRFly and Defects4J were used in our experiments. We observed variance even when the same seeds were used.

To answer this question, we compared all of the RL algorithms with their optimal credit assignment technique that was identified in RQ1 (Table 5). For PM, we can see that 38 unique bugs were patched which is the same as the baseline. However, the success rate of the overall repair attempts was 1.2% lower. As for UCB, only 36 unique bugs were patched, but the overall success rate was 2.7% higher. AP had 37 unique bug patches, one less that the baseline, with a 0.3% decrease in overall success rate. Finally, with the epsilon-greedy experiment 39 unique bugs were patched (1 more than the baseline) with a 4% higher success rate for repair attempts. Given these results, it is evident that in our experiments, the epsilon-greedy algorithm with average credit assignment exhibited the best performance.

Figure 2 shows the trend for the credits assigned to the different mutation operators within the epsilon greedy algorithm. As observed in previous work Petke et al. (2023), the deletion operator performs best and thus receives higher credit values throughout the search.

Since the bugs that were patched with each experiment varied, we had to look at the intersection of the 35 common bugs that were patched in all of the experiments including the baseline in order to conduct an efficiency analysis. The results of the overall comparison are presented in Table 7. We can see that PM with average credit assignment was the only experiment to achieve lower median and average numbers than the baseline for the variant numbers of successful patches. However, it is important to note that the success rate of the experiments varied. Therefore, while the epsilon-greedy experiment does have higher median and average values for the variant number of the patches, this might be due to the higher success rate which skewed the values.

**RQ2**: Epsilon-greedy is the most effective mutation operator selection strategy, while probability matching is the most efficient for heuristic-based APR.

### RQ3: Additional mutation operators

Given that the epsilon-greedy algorithm with the average credit assignment was the best strategy in answering RQ2, we proceed with this technique for experimentation with additional operators.

We run the experiment using 15 PAR templates (recall Sect. 6.1 where we detail that the remaining 3 templates were excluded in our study) and the 3 GenProg mutations for a total of 18 mutation operators. The experiment resulted in a success rate of 35.5% and patches for 47 unique bugs. As for efficiency, the average variant number for variants that resulted in a patch was 129 and the median 83. When comparing with the JaRFly results with 18 arms in Table 6, we can see that our approach did not improve the results of the standard selection strategy. Our approach with 18 arms was able to solve two bugs fewer and the success rate for repair attempts was also 0.8% lower.

**RQ3**: With the addition of 15 arms, reinforcement learning-guided mutation operator selection does not patch more bugs than the standard operator selection strategy.

### RQ4: RL-aided selection performance

The results of RQ3 evidence that a drastic increase in the number of mutation operators lowers the efficacy and efficiency of the approach. To control the number of arms in the RL algorithm, we decided to experiment with separating the PAR templates into groups (details in Sect. 6.3). We additionally experiment with a second type of reward which is based on the fitness of the variant in reference to its direct parent in the mutation step (recall Sect. 3.2.1). Finally, we alter the rate of learning and experiment with two more aggressive approaches for the reinforcement learning. The first approach considers recalculating the mutation operator probabilities after every mutation instead of every generation. The second recalculates the probabilities every generation, but decreases the population within each one. Since our experiments set a bound of 40 on the population size and a bound of 10 on the number of generations, we flip these values and launch repair attempts bound to a population size of 10 over 40 generations.

**Table 6:** RQ2 &RQ3: Results of the uniform operator selection baseline with the 3 GenProg mutations as well as with the additional 15 PAR templates

Mutations	Success rate	Bugs patched	Avg. variant	Med. variant
GenProg	44.3%	38/44	102	52
GenProg and PAR	36.3%	49/59	120	74

**Table 7:** RQ2. Results for the intersection of the 35 common bugs that were patched in all of the experiments

Repair type	Credit assignment	Success rate	Avg. Variant	Med. Variant
Baseline	–	58.6%	105	57.5
**PM**	A	57.9%	97	50.0
UCB	A	64.0%	125	85.5
AP	W	58.7%	99	61.0
epsilon-greedy	A	63.9%	109	76.0

Highest/lowest values, results of our approach in comparison with baselines

**Table 8:** RQ4. Results of mini-experiments on the sample of 5 bugs for 3, 18, and 7 arms

Arms	Generations	Activation	Reward	Success	Bugs patched	Avg. variant	Med. variant
3	10	Every Gen.	Raw	32%	4	176	123
	10	Every Gen.	Relative	32%	4	218	152
	10	Every Mut.	Raw	0%	0	-	-
	40	Every Gen.	Raw	16%	3	158	120
18	10	Every Gen.	Raw	31%	3	116	92
	10	Every Gen.	Relative	31%	4	219	133
	10	Every Mut.	Raw	0%	0	-	-
	40	Every Gen.	Raw	15%	3	175	129
7	**10**	**Every Gen.**	**Raw**	**43%**	**4**	**145**	**108.5**
	**10**	**Every Gen.**	**Relative**	**43%**	**4**	**229**	**186**
	10	Every Mut.	Raw	0%	0	-	-
	40	Every Gen.	Raw	18%	3	178	101.5

Highest/lowest values, results of our approach in comparison with baselines

**Table 9:** RQ4. Results for the epsilon-greedy operator selection algorithm with 7 arms (GenProg and grouped PAR Mutations) with raw/relative fitness run on the 59 bugs

Reward type	Success rate	Bugs patched	Avg. variant	Med. variant
Raw	34.3%	44	123	81
Relative	37.5%	46	176	120

**Table 10:** RQ4. Comparison of quality values, and percentage of patches with 100% quality between our approach and the quality results presented in the JaRFly paper Motwani et al. (2022)

Repair	Min.	Mean	Median	Max.	100% Quality
JaRFly GenProg	64.8%	95.7%	98.4%	100%	24.3%
JaRFly PAR	64.8%	96.1%	98.5%	100%	13.8%
**RL-based APR**	63.5%	95.8%	98.5%	100%	34.0%

Highest/lowest values, results of our approach in comparison with baselines

Table 8 presents the results of the mini-experiments that we ran to test the additional settings of grouped mutations, reward types, and aggressive learning. These experiments were on the same sample of five bugs that was used in the preliminary experiments for tuning the learning rate (recall Sect. 6.3). The results confirm that increasing the number of arms from 3 to 18 does hinder the performance of the approach. However, we can also see that keeping the 18 mutations but grouping them into 7 arms instead does greatly improve the results. The more aggressive learning achieved both through a smaller generation size or adjusting the operator probabilities after every mutation yielded worse results.

From here, we proceeded with additional experimentation on the two most successful experiments (emboldened in Table 8). Table 9 presents the results of these two experiments on the dataset of 59 bugs (64 that were reported as patched using GenProg and PAR in JaRFly minus the 5 we used for the preliminary experiments). The experiment with the fitness relative to the parent as the reward achieved a 3.2% increase in the success rate of the repair attempts and patched 2 additional bugs. Therefore, we decided to test it on the additional 289 bugs that the JaRFly reports to not be able to generate patches for with GenProg and PAR (recall Table 2). Our approach produced 80 patches for 10 bugs from the set that JaRFly didn’t patch. We then reran those 289 bugs for the baseline and found 93 patches for 23 unique bugs. However, after manual investigation of the patches, we found that only 2 of the 93 patches in the baseline were correct and corresponded to 1 unique bug. None of the patches generated using the RL strategy were correct.

We follow the patch evaluation methodology of JaRFly Motwani et al. (2022) to assess the quality of the patches generated by our approach. We assess the quality of 487 patches that correspond to the 51 defects that the epsilon-greedy operator selection algorithm with average credit assignment, relative fitness values as the reward and 7 arms generated on the benchmark including the PAR templates (recall Table 9). Table 10 presents the results of the quality scores of the defects. We remove the 2 patches generated for the Time 19 bug from our analysis as they did not pass any test in the evaluation test suite. The results do not show improvement in the minimum, maximum, median, or average quality values. However, we can see that the percentage of patches that were evaluated to have 100% quality is 34% which is significantly higher than JaRFly.

**RQ4**: Reinforcement learning-aided mutation operator selection is comparable in terms of bugs fixed to the baseline standard uniform selection approach, though we observe more test-passing variants generated.

## Discussion

In this work we explored whether RL-based operator mutation selection leads to more effective and efficient program repair in search-based APR.

Based on our results (recall Sect. 7.2), we deduce that the environment in heuristic-based APR is stationary, i.e., the optimal probabilities for the arms in the RL algorithms do not change over time. The probability matching algorithm had the lowest success rate in our experiments as it is the most simple approach (recall Sect. 3.2). Adaptive pursuit performed slightly better which can be explained by the improvement this algorithm accounts for in the convergence rate. The UCB algorithm improves the success rate even further. This can likely be contributed to the fact that it has been proven to optimise the convergence rate regarding the cumulative gain Auer et al. (2002). However, to do so, the UCB algorithm continues to tune the exploration-exploitation tradeoff as the search progresses. Given that the environment in heuristic-based APR is stationary, we hypothesize that this simply added noise to the learning process and made UCB to over-exploit.

The results demonstrate (Sect. 7.3) that the most effective mutation operator selection strategy (epsilon-greedy) is not the same as the most efficient (PM) which stems from the exploration-exploitation tradeoff. Algorithms that favour exploitation are less effective at finding patches for difficult-to-patch defects since they do not explore the search space as extensively. However, their more exploitative nature will allow them to find the patches for easier-to-patch defects more quickly.

Increasing the number of mutation operators from three to eighteen greatly increased the search space and effectively slowed our approach’s learning rate. However, grouping mutations that target similar defect types together into a single arm representation within the RL algorithm showed an improvement as the power of the PAR fix templates was maintained while controlling the noise that the large number of arms added.

**Fig. 3:**
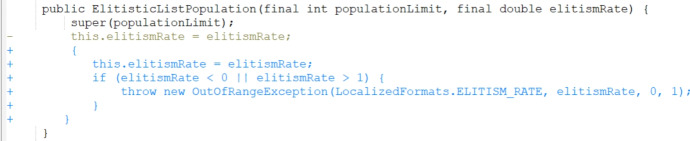
Bug Math 35 patch generated using JarFly with random operator selection

**Fig. 4:**
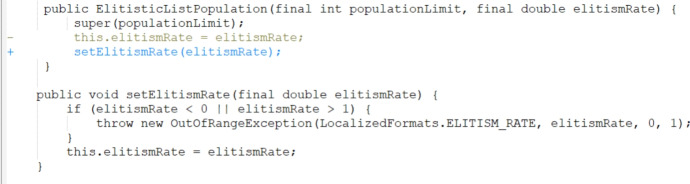
Bug Math 35 Developer-written patch

**Fig. 5:**
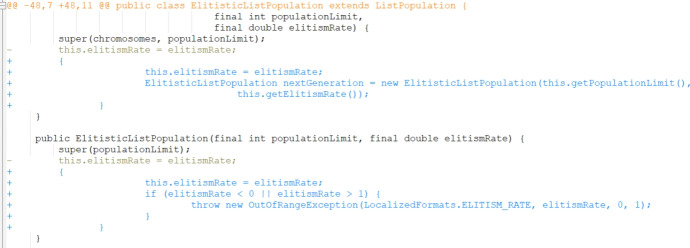
Bug Math 35 patch generated using JarFly with epsilon-greedy guided mutation operator selection

As explained in Sect. 7.5, there were 289 bugs that the baseline reported not to fix in the original paper. We reran the tool on these bugs and manually evaluated the patches generated. Our manual investigation revealed that 2 correct patches were produced and that they correspond to the same bug. We present a successfully generated patch example by the baseline JarFly in Fig. 3 and the corresponding patch written by developers for the same bug in Fig. 4. We can see that instead of a direct call to the setter function with the corresponding argument, the body of the callee function was inserted instead which results in semantically equivalent code. We then present a patch generated by the best RL-guided setup of epsilon-greedy operator selection with average credit assignment, and grouped PAR template mutation operators in Fig. 5. We can see that while the patch does include the correct change, it also adds incorrect code into another part of the code and thus ultimately produces an incorrect patch.

Results show that while the RL approach generated more test-passing variants, it did not significantly improve the number of bugs patched. We hypothesize that this is due to three different reasons. Firstly, since we limit the number of generations to 10 in the experiments, the learning might have been too slow to begin taking effect. Thus, such an approach may only be effective in cases where the search is very long and the probabilities are learned quickly. Secondly, the fitness function in this instance might be too coarse to effectively guide the learning. Finally, the type of edit may not be a sufficient source of information to steer the random edit generation toward the search sub-space containing the correct patch.

Addressing the first theory of extending the time budget is too computationally costly to explore in this work (see Sect. 6.2). We leave this to be explored in future work. We considered exploring the second theory of refining the fitness functions as this is what drastically diffrentiates APR from evolutionary algorithms in other contexts. In the domain of automated program repair, fitness evaluation is frequently binary in nature based on whether a program passes all the tests in a test suite. Previous work has explored a finer-grained approach that accounts for varying failure types showing lack of clear improvement Guizzo et al. (2021); Bian et al. (2021). Given negative results in previous work and computational costs, we decided to not explore this path in this study.

We were most interested in exploring option 3, which is that the type of edit alone is not sufficient to steer the learning. We thus decided to conduct an experiment to explore whether enriching the reinforcement learning algorithm with additional information would yield better results. We implemented functionality to allow the reinforcement learning agent to differentiate between different mutation targets. We consider 3 categories of code locations: special locations (break statement, continue statement, throw statement, variable/array initializer), single-line locations, (all other single statement mutation locations which don’t fall in the “special” category), and multi-line locations (blocks of code such as conditionals and loops). For each mutation, the reinforcement learning agent now has 3 arms, one for each of the location categories. Once a mutation and a location to be mutated is selected, the reinforcement learning agent identifies the category of the location and the type of mutation selected and a reward is given to the relevant arm. With this, we were able to insert context on the code being mutated to the RL agent and further fine-grain the process. We conduct an experiment of this setup on using the previously identified best combination of configuration: epsilon-greedy algorithm with average credit assignment and relative fitness applied every generation. We set the experiment to the 3 standard insert, replace, and delete mutations resulting in 9 arms for the RL algorithm (3 mutation operators x 3 code location categories).

Unfortunately, only 30 out of the 44 bugs were patched with this new set up with a 45.1% success rate, an average of 96 for the successful variant number and a median of 29. When comparing with the results in Table 5, it is clear that this direction is less successful as this combination was previously able to patch 39 bugs with a 48.3% success rate. This might be due to the fact that the additional context added here does not align with the underlying factors that lead to successful patches. The categories of locations added complexity to the reinforcment learning algorithm without providing sufficient correlation to the qualities that contribute to plausible patches. Future work can explore other contextual information that may better guide the learning and search through different or more refined code location categories than the ones that we explore here.

Out of all APR approaches to-date, heuristic-based techniques have been the most adopted in industial settings Kirbas et al. (2020); Haraldsson et al. (2017); Marginean et al. (2019) (with Marginean et al. (2019) using multiple approaches). We pose this is largely due to heuristic-based techniques not requiring a training stage, a pre-trained model, or constraint solvers. We recognise the potential in their continued scaling while accounting for the high success rates of end-to-end learning-based strategies and large language models Sobania et al. (2023); Jiang et al. (2021); Zhu et al. (2021); Xia and Zhang (2022).

## Related work

Our literature review revealed four popular approaches for RL-based mutation selection. None of these, however, have been applied to improve mutation selection strategies for improvement of existing software. We are the first to do so for the problem of APR.

*Mutation operators:* Papers that investigate varying operator probabilities Tuson and Ross (1996); Murata and Ishibuchi (1996) and tuning parameters generally Aleti and Moser (2016); Moazen et al. (2023) in the field of genetic algorithms have been around for decades Stanczak et al. (1999); Julstrom (1997). These works generally focus on the adaptive operator selection based on a probability matching strategy that references the fitness improvements of the individuals Vafaee et al. (2008); Soria-Alcaraz et al. (2014). Thierens et al. propose an alternative to this through their adaptive pursuit strategy Thierens (2005). Other papers focused on the mutation operator specifically both in the field of genetic algorithms Hesser and Mäinner (1991); Ali and Brohi (2013) and genetic programming Anik and Ahmed (2013); Hong et al. (2014). The shortcomings of mutation operation, particularly in promoting diversity for GP Jackson (2011) are discussed in the literature as well. Glickman et al. Glickman et al. (2000) and Friedrich et al. Friedrich et al. (2018) address issues such as premature convergence and local optima escape.

Multiple studies alter operator probabilities using reinforcement learning Yu and Lu (2023); Yu and Zhou (2023); Yu et al. (2024). These studies address both credit assignment strategies as well as operator selection techniques, therefore they are particularly relevant to our paper Awad et al. (2022); Li et al. (2023); Zhang et al. (2023). More specifically, extensive research has been done on operator selection strategies that use multi-armed bandits Costa et al. (2008); Fialho et al. (2009, 2010); Thierens (2005); Murata and Ishibuchi (1996); Eiben et al. (2007); Pettinger (2002); Maturana et al. (2009). These paper mainly focus on augmenting evolution strategies in the context of numerical optimisation.

In the field of APR specifically, we found some work by Le Goues et al. Le Goues et al. (2012) on operator design choices. Smigielska et al. Smigielska et al. (2021) propose a uniform strategy when choosing operators. Enhancements were suggested using probabilistic models Soto and Le Goues (2018), program context Wen et al. (2018); Ullah et al. (2023), and bug fix history Le et al. (2016). Soto et al. Soto (2019) further propose a set of techniques for enhancing the quality of patches. All of this work focuses on a fixed distribution of probabilities for operator selection. Our work takes this a step further by using reinforcement learning to modify this distribution during search.

*Machine learning for APR:* The utility of the machine learning component varies from predicting patch correctness Schramm (2017); Tian et al. (2023), to predicting the type of fault Valueian et al. (2022), to predicting whether continuing the search process will result in a repair Le (2016). Existing work guides the search process by learning from code patterns and features Chen et al. (2022); Valueian et al. (2022), bug reports Liu et al. (2013), program namespace Parasaram et al. (2023), or context and statistics Jiang et al. (2019); Yu et al. (2023). Ji et al. Ji et al. (2022) applies program synthesis to the problem of automated program repair. Conner et al. Connor et al. (2022) use neural machine translation models that generate edit operations for patching bugs instead of translating from the buggy to fixed source code directly. Additional work focuses on more specific types of repair, e.g., for conditional statements Gopinath et al. (2016), compilation errors Ahmed et al. (2022), API misuses Wu et al. (2022), or specific program languages Lajko et al. (2022). Finally, more recent work also include repair based on generative language models Kang and Yoo (2023); Xia et al. (2023) as well as more interactive user-centric techniques Liu et al. (2024); Geethal et al. (2023).

## Threats to validity

*External threats:* Defects4J is composed of real-world bugs and is a widely used benchmark in the literature. However, there always remains a threat of generalisability. Moreover, our methodology uses Java which may not generalise to other programming languages. Our choices of benchmark and language enable direct comparison with state-of-the-art approaches. We do not limit our approach to any specific benchmark, programming language, or tool therefore these threats can be mitigated through additional implementations.

*Internal threats:* Our experiments were conducted on a cluster of computation nodes with varying specifications. We mitigated this threat by comparing the variant number of the successful patch instead of execution time to measure efficiency.

*Threats to construct validity:* The results of the JaRFly baseline were not reproduced despite using the same search parameters. Since our approach uses the most recent version of both JaRFly and Defects4J, the discrepancy is likely due to a version issue, the heuristic nature of search, and the omission of 3 of the PAR templates as explained in Sect. 6.1. Patch quality assessment remains an open question in the field. We mitigate the threat of potential overfitting by evaluating the patches on held-out evaluation test suites, and by manual evaluation of final test-passing variants.

## Conclusions and future work

In this work, we introduce a reinforcement learning approach for mutation operator selection in heuristic-based automated program repair. We conducted an extensive empirical evaluation, spanning four learning algorithms and two credit assignment techniques, and assessed the effect of reward types, number of mutation operators, and activation points on the performance. Our findings reveal that this approach is comparable in terms of bugs fixed to the baseline standard uniform selection approach, despite RL-based selection generating more test-passing variants.

However, our analysis suggests several avenues for future investigation. Firstly, the RL mechanism may not have sufficient time to significantly influence the probability of generating the correct edit within our budget-limited experiment. Secondly, the fitness of variants may not serve as the most efficient reward source during learning. Lastly, uncertainty remains regarding the effectiveness of only using edit types to steer the search towards correct solutions.

In summary, our study provides valuable insights into RL-aided mutation operator selection whilst also underscoring the complexity inherent to automated program repair. Future research efforts should strive to address these challenges, through innovative methodologies that balance computational efficiency with robustness in identifying correct patches.

## Data Availability

All source code, supplementary materials, and instructions needed to replicate our results are publicly available at: https://github.com/carolhanna01/jarFly-learner/tree/operator-selection. Results available separately on Zenodo at: https://zenodo.org/records/7579947.

## Footnotes

1 Details about the papers found are presented in Related Work (Sect. 9).

2 Refer to our tool comparison report for full details: https://github.com/carolhanna01/jarFly-learner/blob/operator-selection/reports/Tools.pdf

3 The rest of the plots for PM, AP, and UCB can be found in the project’s repository.
